# Exploring Factors Associated with Functional Change and Predictors of Participation Improvement—A Two Years Follow-Up on People with Depression

**DOI:** 10.3390/ijerph18073439

**Published:** 2021-03-26

**Authors:** Wen-Chou Chi, Chia-Feng Yen, Tsan-Hon Liou, Kwang-Hwa Chang, Hua-Fang Liao, Ya-Li Chang

**Affiliations:** 1 Department of Occupational Therapy, Chung Shan Medical University, Taichung 40201, Taiwan; y6312002@gmail.com; 2 Occupational Therapy Room, Chung Shan Medical University Hospital, Taichung 40201, Taiwan; 3 Department of Public Health, Tzu Chi University, Hualien 97004, Taiwan; mapleyeng@gmail.com; 4 Department of Physical Medicine and Rehabilitation, Shuang Ho Hospital, Taipei Medical University, Taipei 11031, Taiwan; peter_liou@shh.org.tw; 5 Department of Physical Medicine and Rehabilitation, School of Medicine, College of Medicine, Taipei Medical University, Taipei 11031, Taiwan; 6 Graduate Institute of Injury Prevention and Control, Taipei Medical University, Taipei 11031, Taiwan; chang2773@gmail.com; 7 Department of Physical Medicine and Rehabilitation, Wan Fang Hospital, Taipei Medical University, Taipei 11696, Taiwan; 8 School and Graduate Institute of Physical Therapy, National Taiwan University, Taipei 10055, Taiwan; hfliao@ntu.edu.tw; 9 Department of Psychiatry, Chung Shan Medical University Hospital, Taichung 40201, Taiwan

**Keywords:** social participation, depression, WHODAS 2.0, disability, functioning, ICF

## Abstract

The purpose of this study is to understand the functional status distribution and to explore the factors associated with changes in functional status and social participation in people with depression using two-year follow-up data. Subjects were selected from the Taiwan Databank of Persons with Disabilities (TDPD) if they had an evaluation date between July 2012 and 31 December 2017. We used data for 1138 individuals with multiple evaluation records and who were diagnosed with depression. The WHO Disability Assessment Schedule 2.0 (WHODAS 2.0) was the primary functional status measure. Other factors selected from the TDPD included social demographic data, living situation, employment status, economic status, and educational level. The results show scores in all dimensions of the WHODAS 2.0 declined over two years, especially in the domains of cognition, household activities, social participation, and total WHODAS 2.0 score. Aging groups showed poor recovery in cognition, getting along with others, and household activities. People living in suburban areas showed poorer recovery than people living in rural and urban areas in cognition, self-care, and general function (total score of WHODAS 2.0). Employment was also strongly associated with functional recovery in household activities, social participation, and general function. The original scores for cognition and getting along with others showed a significant negative relationship with social participation improvement. Our results can be used by policy makers to provide resources and conduct investigations, and by clinicians when making rehabilitation plans.

## 1. Introduction

Depression is a common mental disorder. In 2015, its worldwide prevalence was estimated to be 4.4%, affecting 322 million people [1]. This affliction introduces several problems, such us decreasing quality of life, increasing the suicide rates, and presents as a disability [2].

People with depression may suffer from diverse symptoms affecting human function. These symptoms can include negative moods, such us sadness, a lack of interest or pleasure, and low energy. According to the 11th revision of the International Classification of Diseases (ICD-11; [3]), depression symptoms are mainly depressive mood or depressive mood accompanied by a loss of pleasure, which adversely impacts individual social functioning. These symptoms cause more severe functional limitations in life situations such as interpersonal relationships [4], and reduced cognitive [5], psychosocial [6], and physical functioning [7], and a diminished ability to complete the activities of daily living [8]. Such changes affect people’s interactions with their environment, which is termed participation [9].

Social participation is a major function and health index for humans, and is defined as involvement in life situations [10]. Specifically, “active involvement in activities that are intrinsically social and occur in a societally defined context” [9]. Social participation can be divided into six non-discrete levels of participation according to different goals: (1) preparing for activities to connect with others, (2) getting along with others, (3) interacting with others without performing specific activities together, (4) cooperating with others to achieve a common goal, (5) helping others, and (6) contributing to society [11]. People with depression are limited by their symptoms and most struggle to engage socially. For example, a loss of interest can prevent people with depression from reaching the above goals and impacts their quality of life [12]. Therefore, improving social participation functioning is an important goal when helping people with depression.

Functioning, including social participation, in people with depression may decrease, remain unchanged, or improve over time [6,13]. Such changes depend on various factors and situations. For example, gender and age may affect the functional level of the activity of people suffering from depression [14,15,16]. Further, where people live is also related to their daily functioning. For example, big cities and non-instrument living are thought to be better for people with depression [16,17]. A higher education status and employment, which can lead to more opportunities to interact in social environments, can improve depression symptoms and social function [18]. However, these studies lack follow-up data and most focus on specific populations.

People with depression are limited by their symptoms, and most have difficulty engaging in social participation. Increasing social participation functioning is therefore an important goal when helping people with depression.

In our study, we use a large database and longitudinal data to explore (1) the functional status of people with depression, (2) the factors associated with functional changes in people with depression over a two-year period, and (3) the factors that can be used to predict improved social participation.

## 2. Materials and Methods

### 2.1. Participants and Data

Our study used secondary longitudinal data collected from the Taiwan Databank of Persons with Disabilities (TDPD) from subjects with type 1 disability (mental function) from July 2012 to December 2017. This study was approved by the institutional review board of Hualien Tzu Chi Hospital (IRB-107-46-B).

Subjects were included if they were diagnosed with disruptive mood dysregulation disorder, major depressive disorder, persistent depressive disorder/dysthymia, premenstrual dysphoric disorder, substance/medication-induced depressive disorder, depressive disorder due to another medical condition, other specified depressive disorders, or an unspecified depressive disorder. There were 4776 cases that matched these criteria.

**Figure 1:**
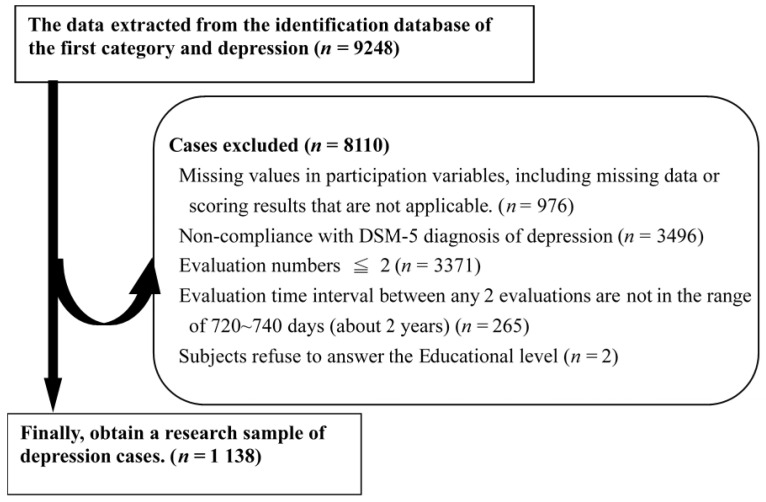
The selection and exclusion process of participants.

Functional improvement takes more than a year to see visible change [19]. Therefore, we selected individuals who had been evaluated at least three times (T1, T2, and T3), with an interval of 720–740 days between any of the three evaluation time points (T1 to T2, T2 to T3, or T1 to T3). We analyzed data using two evaluation points (T1 to T2, T2 to T3, or T1 to T3) of each subject. If the intervals between T1 to T2 and T2 to T3 were both between 720 and 740 days, only T1 and T2 data from those participants were included in our analyses. In total, 1138 subjects met these criteria and were included in the study (Figure 1).

The TDPD was created as part of a system to determine eligibility for disability welfare support in Taiwan. The database contains a wide range of data, but does not include medical records due to the purpose of the database is not for medical use. We chose to analyze the following factors based on our literature review and study aims: the WHO Disability Assessment Schedule 2.0 (WHODAS 2.0); the urbanization level of the areas where subjects lived; and their employment status, economic status, education level, age, gender, and residence type.

The WHODAS 2.0 measures functioning and disability and was developed according to the International Classification of Functioning, Disability, and Health (ICF). The WHODAS2.0 can assess people with any condition [20,21,22]. The Taiwanese version shows good reliability and validity [23]. The 32-item version of the WHODAS 2.0 includes six domains: cognition, mobility, self-care, getting along with others, life activities (household), and social participation. Each domain has its own score, from 0 to 100, where the higher the score, the poorer the functioning. In addition to the six domains, the WHODAS2.0 contains a summary score, which is the total score of the six domains, and a general score of functional disability from 0 to 100. Education level was classified as college and above, senior high school, junior high school, primary school, and no formal education. Urbanization level was classified as rural, suburban, or urban. Regarding residence type, subjects were classified as community-dwelling or institutionalized.

### 2.2. Analysis

We performed statistical analyses using SPSS software. We analyzed demographic characteristics, and used *t*-tests to compare initial evaluations with the evaluations after two years for five of the WHODAS 2.0 domains and the total score. We excluded the self-care domain from the analysis because the data were skewed and showed high kurtosis.

We performed a regression analysis using change in social participation over two years as the dependent variable, and age, gender, residence type, urbanization level, and the initial evaluation scores for cognition, mobility, getting along with others, and life activities (household) as independent variables.

## 3. Results

The majority of subjects were female (73%) and most were unemployed (14.6% were employed; Table 1). Almost all subjects (97.5%) lived within their communities. Only 26.2% had an education level of senior high school or higher (more than nine years of education). Almost half of the subjects were 45–60 years old and, within this age range, 18.1% of subjects were 45–50 years old, 16.3% were 50–55 years old, and 15.4% were 55–60 years old. The WHODAS scores varied significantly between genders, among age groups, and according to employment status. Female subjects had lower WHODAS scores than male subjects and unemployed subjects had lower WHODAS scores than employed subjects. Regarding age groups, people above 35 years old were more common than younger groups.

All domain scores decreased between pairs of evaluations, indicating amelioration over time. In particular, cognition, getting along with others, household activities, and social participation functions improved significantly (Table 2). Changes in cognition, getting along with others, and household activities differed among age groups. However, unlike the younger age groups, subjects over 65 years old showed no improvement.

When stratified by the urbanization level, there were significant differences in cognition, self-care, and summary scores: subjects living in suburban areas showed less improvement in these areas. When stratified by employment status, there were significant differences in household activities, social participation, and summary scores: unemployed subjects showed greater improvement than employed subjects (Table 3).

The regression model showed an R^2^ of 0.11 and explained a significant amount of the variance in the data (*p* < 0.01). We found that only cognition and getting along with others domains could predict changes in social participation. Poorer scores in these domains upon first evaluation were associated with a weaker recovery in social participation (Table 4).

## 4. Discussion

We conducted a cohort study using data from the TDPD to understand the functional changes in people with depression over two years and identify the factors affecting social participation. From the participant sample distribution, we found depression was more common in middle-aged people and in women, which is consistent with most previous studies [24,25]. Most subjects did not have a job at the time of their first assessment. We speculated that the main reason for this is that their condition was already relatively serious when they obtained their certificate of physical and mental disability. Despite this, most subjects lived within their communities rather than in institutions; this corroborates with current global trends in deinstitutionalization of people with disabilities. The overall functioning of people with depression improved within two years, which is consistent with results of other studies [6,13,19]. Their functioning is poorer than that of people without depression [6,13], but, after a period of time, people gradually recover from depression.

The second important finding of this study is that some functions have the potential to recover, while others do not. The most obvious improvements were in cognition, household activities, getting along with others, and social participation. However, mobility showed no significant change. Improved cognitive function can be expected when depression symptoms improve, since depression symptoms seriously affect cognitive function [5]. We speculate that improved social participation and getting along with others are also related to ameliorating of symptoms, since this permits people with depression to go out and interact with others. The improvement in the household domain may be related to sample distribution. Almost all subjects were community-dwelling, meaning they interacted with household activities in their everyday lives. There was no significant change in mobility in this study. Mobility was not a major problem for this cohort (WHODAS 2.0 score of 22 points at the initial evaluation).

Thirdly, we found improved functioning differed according to age group and place of residence. Subjects over 65 years old showed little recovery in functioning, and significant degeneration in cognition, household activities, and getting along with others. Older adults with reduced athletic ability and physical functioning are at increased risk of social rejection and loneliness, since it is difficult for them to participate in community activities, maintain friendships, and visit family and friends [26]. Accordingly, functional degradation is expected. Place of residence affected cognition, self-care, and the WHODAS 2.0 summary score. Among these factors, subjects living in suburban areas showed the least improvement, and regressed in self-care. Suburbanization is a population shift from central urban areas into suburbs, which can form of (sub)urban sprawl [27]. Most people who live in the suburbs are part of the working population and work in an urban city. These suburbs are designed for this working population. Most members of our study cohort were unemployed, and living in the suburbs may not fulfill the needs of unemployed people with depression. Combined, these considerations may help explain our results.

Finally, we showed cognitive function and the ability to get along with others can predict the degree of improvement in social participation after two years. There is a clear correlation between cognitive function and depressive symptoms. Studies have shown that cognitive impairment in patients with major depressive disorders includes, along with other issues, memory decline [8], impaired executive function, and slower processing speeds compared with people without depression [5]. The more serious these issues, the greater their impact on the recovery of social participation. In the early stages of depression, it may be important for the individual to focus on maintaining cognitive functioning. Participation is linked to the environment, including interactions with people [10]. Therefore, getting along with others is a major contributor to social participation. Our results support this strong relationship.

The samples in our study were taken from the TDPD database, which provides secondary data. One limitation of our study is that it was not possible to determine the time of onset of depression for each case. In addition, the severity of depression could not be determined from the data. The second limitation is that the TDPD is a national database containing limited information. The variables used in our study were constrained by the database and contacting subjects was impossible due to subject anonymity. The third limitation is the model’s R^2^ of 0.11, which infers that other factors can predict the functional recovery of social participation, e.g., psychotherapy [28]. Future studies may address different factors.

Our results provide important evidence for informing policy. When developing systems to prevent depression and to support people that have depression, policy makers can use our results to direct more resources toward aging groups, as older adults with depression often have a poor prognosis. Further, greater support of systematic investments in suburban areas is needed, especially for care support and community rehabilitation centers. Employment policy makers could investigate the factors that result in the worsening function of employed individuals with depression and could work to improve the outcomes in that area. Here, we proposed factors associated with improving social participation, and clinicians can use this information to devise treatment plans. The “cognition” and “getting along with others” factors can be used as grouping indexes or training targets for rehabilitation purposes. When evaluating these cases, the general prognoses described here will help evaluators explain their results and inform future plans and goal setting.

## 5. Conclusions

Depression is a disorder with far-reaching and significant personal, familial, and social impacts. We conducted a longitudinal study using a large sample size dataset that focused on daily functioning. We provided a systematic analysis of functional changes over two years in people with depression and identified factors that affect these differences, which may help improve care systems for people with depression. Future research should include a more detailed analysis of changes in activities and social participation of patients with depression. Further, focusing on different levels of depression severity and different age groups can help develop better individual care and treatment strategies. There are still many factors associated with improving social participation that require further research to dissect.

## Figures and Tables

**Table 1:** Sociodemographic characteristics of depressive patients in Taiwan (*n* = 1138).

Variables	*n* (%)	WHODAS2.0 Summary Score (32 Items)
		Mean (SD)
Gender **		
Male	307 (27.0)	34.27 (17.50)
Female	831 (73.0)	38.14 (19.10)
Age (years) *		
<30	50 (4.4)	30.19 (16.07)
30–35	58 (5.1)	29.03 (16.94)
35–40	109 (9.6)	36.15 (17.59)
40–45	150 (13.2)	38.54 (18.49)
45–50	206 (18.1)	37.92 (19.32)
50–55	185 (16.3)	38.54 (20.11)
55–60	175 (15.4)	37.99 (18.73)
60–65	126 (11.1)	38.13 (19.09)
>65	79 (6.9)	36.77 (16.79)
**Urbanization level**		
Rural	141 (12.4)	35.47 (19.46)
Suburban	353 (31.0)	35.58 (18.46)
Urban	644 (56.6)	38.35 (18.71)
**Residence** **type**		
Community dwelling	1110 (97.5)	37.07 (18.71)
Institution	28 (2.5)	39.97 (21.58)
**Employment** status ***		
Employed	166 (14.6)	30.81 (16.85)
Unemployed	972 (85.4)	38.22 (18.87)
**E** **conomic status**		
Average	1095 (96.2)	37.09 (18.56)
Middle low and low	43 (3.8)	38.20 (23.50)
**Education** **level**		
Above college	50 (4.4)	35.94 (20.41)
Senior high	248 (21.8)	36.75 (19.69)
Junior high	562 (49.4)	36.52 (18.17)
Primary	249 (21.9)	38.18 (19.07)
No formal education	29 (2.5)	44.72 (15.51)

* *p* < 0.05, ** *p* < 0.01, *** *p* < 0.001.

**Table 2:** The two years difference in performance on the WHODAS2.0 in patients with depression (*n* = 1138).

Domain	Initial Score	Score after Two Years	Significant
	Mean (SD)	Mean (SD)	
Cognition	38.81 (22.98)	35.15 (22.02)	***
Mobility	22.11 (23.49)	20.82 (22.78)	
Self-care	12.14 (17.95)	11.16 (16.74)	+
Getting along with others	50.13 (25.88)	48.34 (24.40)	*
Household	66.02 (41.82)	39.48 (29.30)	***
Social participation	50.22 (32.81)	45.98 (31.29)	***
Summary Score (32 items)	37.13 (18.76)	34.29 (18.09)	***

* *p* < 0.05, *** *p* < 0.001. +: The self-care data were not normally distributed, so a paired *t* test was not performed.

**Table 3:** The two years difference in score on the WHODAS2.0 (T2-T1 or T3-T2 or T3-T1) according to different variables (mean, *n* = 1138).

Variables	Value	Cognition	Mobility	Self-Care	Getting along with Others	Household	Social Participation	Summary Score
Gender								
	Male	−2.38	−0.20	−0.75	−0.33	−26.79	−2.85	−1.56
	Female	−4.13	−1.70	−1.07	−2.39	−26.65	−4.75	−3.28
Age (years)		*			*	**		
	<30	2.09	−1.25	2.06	−0.33	−33.25	−3.50	−1.01
	30–35	−5.78	0.43	−2.07	−3.22	−30.71	−3.88	−2.07
	35–40	−3.81	−0.45	−1.56	−4.36	−29.41	−5.73	−3.22
	40–45	−0.07	−1.92	−2.60	−1.11	−24.66	−5.33	−2.07
	45–50	−4.30	−1.35	−1.55	−4.68	−30.68	−4.00	−3.41
	50–55	−5.57	−3.28	−1.73	−0.72	−28.00	−5.14	−3.10
	55–60	−6.77	−2.14	0.86	−0.81	−30.17	−1.29	−4.07
	60–65	−5.04	0.84	−0.40	−5.36	−27.44	−7.14	−4.78
	>65	2.41	0.63	−0.38	8.43	3.29	−1.27	2.72
Urbanization level		*		**				*
	Rural	−4.15	−1.38	−3.19	−2.07	−22.63	−6.38	−3.02
	Suburban	−0.95	0.34	1.81	−1.16	−26.01	−2.41	−0.73
	Urban	−5.03	−2.18	−2.03	−2.15	−27.93	−4.77	−3.97
Residence type								
	Community dwelling	−3.64	−1.22	−0.90	−1.75	−26.49	−4.26	−2.81
	Institution	−4.46	−4.46	−4.29	−5.36	−34.71	−3.57	−4.50
Employment status						***	**	**
	Employment	−0.72	0.91	−0.60	1.31	2.95	4.37	0.83
	Unemployment	−4.16	−1.67	−1.05	−2.37	−31.69	−5.71	−3.47
Economic status								
	Average	−3.60	−1.21	−0.95	−1.71	−26.42	−4.20	−2.76
	Middle low and low	−5.12	−3.63	−1.86	−5.04	−33.55	−5.23	−5.08
Education level								
	Above college	−7.10	−2.86	−1.00	−2.50	−35.77	−3.50	−5.76
	Senior high	−2.04	−1.39	−1.05	−1.51	−27.20	−5.54	−2.34
	Junior high	−4.45	−0.92	−0.44	−2.40	−28.25	−3.07	−2.73
	Primary	−2.69	−2.16	−1.81	−1.24	−21.05	−5.72	−3.03
	No formal education	−4.48	2.16	−3.79	2.30	−25.17	−4.31	−3.04

* *p* < 0.05, ** *p* < 0.01, *** *p* < 0.001.

**Table 4:** Results from regression analysis showing factors influencing changes in participation using sociodemographic characteristics (age and gender), social factors (urbanization level and residence type), and disability function (cognition, mobility, getting along with others, and household activities).

Variables	Value	Social Participation in Change	
		*p* Value	Standardized Coefficient Beta
Sociodemographic characteristic	Female_Male	0.936	0.002
	30–35_<30	0.712	−0.015
	35–40_<30	0.777	−0.014
	40–45_<30	0.949	0.003
	45–50_<30	0.750	0.019
	50–55_<30	0.969	−0.002
	55–60_<30	0.646	0.026
	60–65_<30	0.623	−0.025
	>65_<30	0.905	−0.005
Social factors	Suburban_Rural	0.149	0.064
	Urban_Rural	0.220	0.054
	Community dwelling_Institution	0.987	0.000
Disability function	Cognition	0.002 **	−0.129
	Mobility	0.867	−0.006
	Getting along with others	0.000 ***	−0.221
	Household	0.115	−0.046

* *p* < 0.05, ** *p* < 0.01, *** *p* < 0.001.

## Data Availability

Restrictions apply to the availability of these data. Data was obtained from Ministry of Health and Welfare and are available from T.-H.L. with the permission of Ministry of Health and Welfare.
